# Synergy of crude enzyme cocktail from cold-adapted *Cladosporium cladosporioides* Ch2-2 with commercial xylanase achieving high sugars yield at low cost

**DOI:** 10.1186/s13068-014-0130-x

**Published:** 2014-09-10

**Authors:** Lei Ji, Jinshui Yang, Hua Fan, Yi Yang, Baozhen Li, Xuejian Yu, Ning Zhu, Hongli Yuan

**Affiliations:** State Key Laboratory of Agrobiotechnology, MOA Key Laboratory of Soil Microbiology and National Energy R & D Center for Non-food Biomass, College of Biological Sciences, China Agricultural University, Beijing, 100193 China

**Keywords:** *Cladosporium cladosporioides*, Cold adaptation, Laccase, MIP, Polysaccharide hydrolases, Synergy, Commercial xylanase

## Abstract

**Background:**

The efficiency and cost of current lignocellulosic enzymes still limit the large-scale production of cellulosic ethanol in industry. Residual lignin after pretreatment severely depresses the activity of polysaccharide hydrolases and the h ydrolysis of holocellulose. If we include in hydrolase mixture construction the ligninase involved in lignin degradation, which mainly includes laccase, manganese peroxidases (MnP) and lignin peroxidase (LiP), it is feasible that this could greatly improve the fermentable sugars yield.

**Results:**

The psychrophilic lignocellulosic enzymes system of *Cladosporium cladosporioides* Ch2-2 including ligninase and polysaccharide hydrolases was suitable for selective delignification and efficient saccharification of biomass with wide thermal adaptability. The purified laccase was optimally active at 15°C and pH 3.5, exhibiting high thermostability over a broad range of temperatures (between 4 and 40°C). In addition, manganese-independent peroxidase (MIP), a special type of ligninase with the capacity to oxidize dimethyl phthalate (DMP) in the absence of H_2_O_2_ and Mn^2+^, was optimally active at 20°C and pH 2.5, exhibiting high thermostability over a broad range of temperatures (4 and 28°C), while depressed completely by Fe^2+^ and essentially unaffected by EDTA. Synergy between Ch2-2 crude enzymes and commercial xylanase obviously enhanced biomass hydrolysis, which could take the place of expensive commercial cellulase mixture. The maximum value of synergistic degree reached 4.7 at 28°C, resulting in 10.1 mg/mL reducing sugars.

**Conclusions:**

The psychrophilic enzymes system of *C. cladosporioides* Ch2-2 with a different synergistic mechanism has huge potential for the enhancement of biomass hydrolysis at mesophilic and low temperatures. The application scope of the lignocellulosic enzyme cocktail could be greatly enlarged by optimizing the operation conditions specific to the characteristics of ligninase.

## Background

Making full use of biomass such as agricultural waste and energy crops to produce clean fuel ethanol on an industrial scale is beneficial to solve the crisis of environmental pollution and energy shortage. Sugarcane bagasse is one of the most abundant byproducts of agroindustry in south China as well as the world [1]. Meanwhile, Jerusalem artichoke is a typical example of the energy crops which can grow in marginal lands, including saline soils and sandy soils, owing to its unique agronomic traits such as tolerance to salt and drought stresses, and thus does not compete for arable lands with grain crops [2]. As a sustainable feedstock, bagasse and Jerusalem artichoke stalks are both considered to be applicable for the large-scale biological production of cellulosic ethanol and other bio-based chemicals. However, the low efficiency and high cost of existing enzymes converting lignocellulose to fermentable sugars still limit the large-scale industrialization of cellulosic ethanol at present. In this respect, the synergistic action of lignocellulosic enzymes has been recognized as a possible way to improve sugars yield at lower enzyme loadings [3].

Synergy between cellulase and xylanase has been extensively studied in prior research [3]. Hu *et al*. showed that cellulose and xylan hydrolysis occurred three times faster when adding an optimized mixture of commercial cellulase and xylanase instead of equivalent cellulase [4]. The synergistic interaction between endo-xylanase from family GH10 and xyloglucanase from family GH5 also enhanced the hydrolytic activity of commercial cellulase over a range of pretreated lignocellulosic substrates [5]. Additionally, Schilling *et al*. found that the significant increase in saccharification efficiency through xylanase-aided synergism was not limited to same-species enzyme sources [6]. However, these synergistic reactions were almost held at 50°C or thereabout, according to the optimum temperature of cellulase and xylanase [7], which could not fully meet the demands for application at environment temperatures.

However, synergistic cooperation between ligninase and polysaccharide hydrolases during biomass degradation has been largely ignored. Due to the complexity of the structure of the plant cell wall, lignin is hard to totally remove after pretreatment with current methods [8]. The hydrolase activities and total sugars yield are severely reduced by the residual lignin component and its related derivatives. It was showed that various cellulases and xylanases differed by up to 3.5-fold and 1.7-fold respectively in their inhibition by lignin [9]. Grabber *et al*. indicated that reduction of lignin content or ferulate-lignin crosslinking would improve the fermentation of cellulose and hemicellulose more than current approaches for shifting lignin composition [10]. In a recent study, a higher reducing sugars conversion (60%) was achieved when adding commercial laccase into a mixture of cellulases and hemicellulases at 45°C [11]. Therefore, ligninase should be taken into account in enzyme cocktail construction for saccharification in order to maximize the hydrolysis efficiency.

One special kind of ligninase MIP receiving little attention in recent years may play a different role in lignin degradation [12,13]. It was first reported in the extracellular fluid of the white-rot fungus *Bjerkandera* sp. BOS55 as having the capacity to oxidize DMP in the absence of H_2_O_2_ [12]. In contrast with MnP, MIP was not inhibited by 1 mM EDTA and was not stimulated by Mn^2+^ Mn^2+^ addition [14–16]. In view of its simpler substrate oxidation mode, the function of MIP in the complex synergistic system of lignocellulosic enzymes still needs to be clarified.

Lignocellulosic fungus *Cladosporium cladosporioides* was reported to secrete laccase, MnP, LiP, cellulases and hemicellulases. Several laccases have been purified from the culture supernatant of *C. cladosporioides*, which usually showed relative activities over a range of pH 3 to 6 and 40 to 70°C [17,18]. Previous research of *C. cladosporioides* mostly focused on its good capacity for dye decolorization and various enzyme activities in mesophilic temperatures [7,19,20]. There was only one report about psychrophilic *C. cladosporioides*, which was isolated from Antarctica at either 4°C or 15°C with the ability to produce endoglucanase [21]. Further studies about psychrotolerant *C. cladosporioides* secreting ligninase and polysaccharide hydrolases in biomass degradation will considerably enlarge its application range.

Cold-adapted microorganisms have a considerable potential in biotechnological application, including waste treatment and bioremediation at ambient temperatures, textile and food industries [22,23]. Bioconversion catalyzed by cold-adaptive enzymes produced by these psychrophilic microorganisms have three advantages, firstly they have the potential to economize processes by saving energy because of their high activity at low and moderate temperatures and thus offer potential economic and environmental benefits [23]. Secondly, mild industrial conditions at an ambient temperature are beneficial for preventing modification of original heat-sensitive substrates and generation of adverse by-products [24]. Thirdly, the application of psychrophilic enzymes will make the industrial operation more convenient and safer, which is a tendency in traditional industry [24].

In our previous work a lignin-degrading psychrophilic fungus strain *C. cladosporioides* Ch2-2 was isolated from Changbai Mountain (China) forest soil, which was able to remove basic dye effectively [25]. In this study the lignocellulose degradation characteristic of psychrophilic *C. cladosporioides* secreting laccase, MIP, cellulases and hemicellulases will be investigated for the first time. According to the cold-adapted property of purified laccase and MIP, hydrolysis was greatly enhanced at either temperate or low temperature through synergy between Ch2-2 crude enzymes and commercial xylanase. The results will provide new perspectives to create lignocellulosic enzyme cocktails with a lower cost and higher efficiency.

## Results

### Psychrophilic adaptability of *C. cladosporioides* Ch2-2

**Figure 1:**
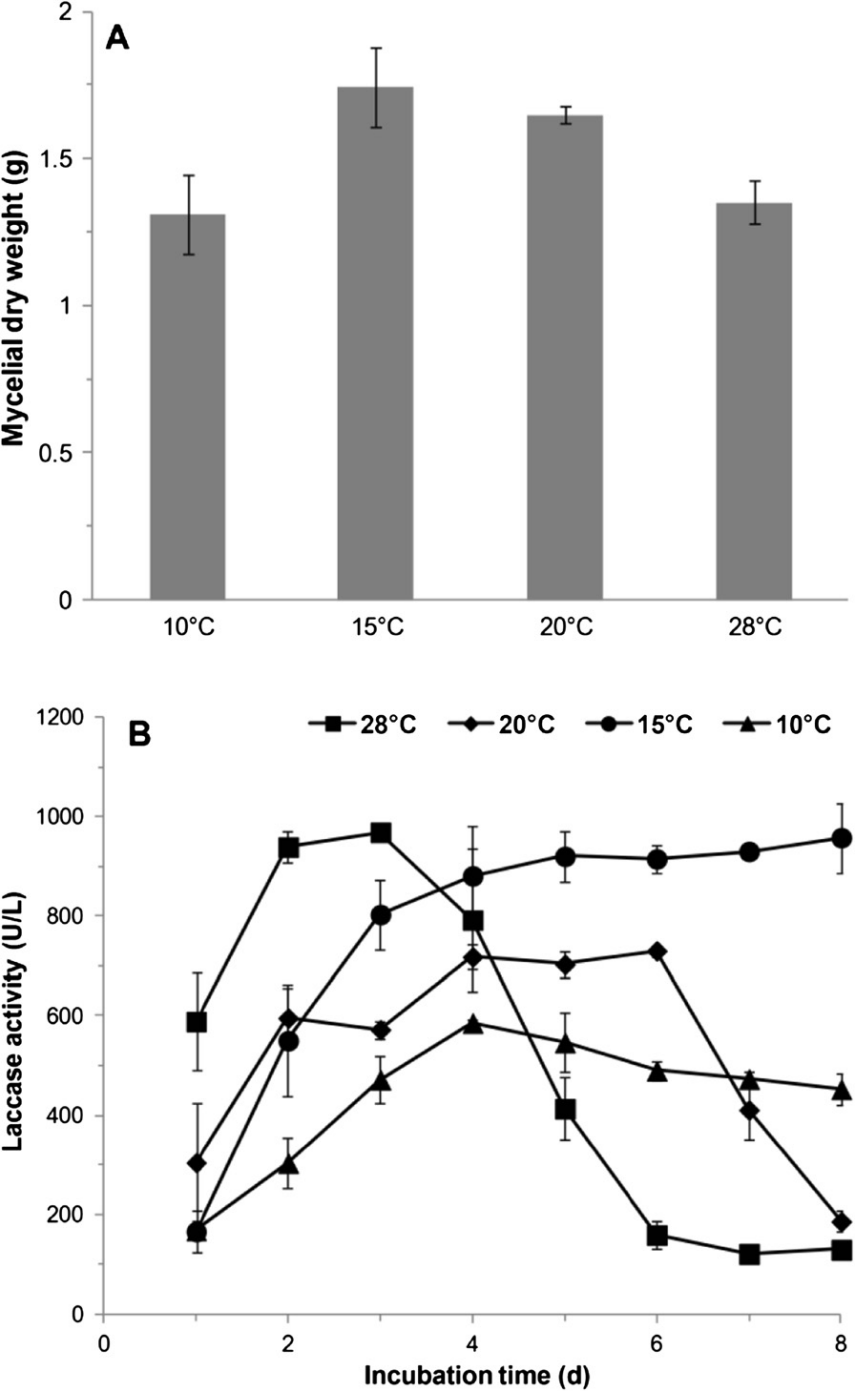
**Effects of different temperatures on growth (A) and laccase production (B) of** ***C. cladosporioides*** **Ch2-2 after 7 days incubation.**

*C. cladosporioides* Ch2-2 was cultured in GPY medium under different temperatures for 7 days separately, and then dry weights were measured. Data showed that Ch2-2 was able to keep almost the same high dry weight (1.31 to 1.74 g) in the temperature range from 10°C to 28°C while the optimal growth temperature was 15°C (1.74 g) (Figure 1A). It indicated that Ch2-2 was a psychrophilic fungus with wild thermal adaptability, whether in normal or in low temperature.

Along with the growth of Ch2-2, laccase activity was checked, which was reported to be connected with decolorization of basic dye [25]. As shown, from the first day of incubation laccase was secreted, whether in low temperature (10°C) or in normal temperature (28°C) (Figure 1B). In accordance with the optimal growth temperature, 15°C was found to be best for the stability of laccase production. It could reach almost the maximum (920 U/L) on the fourth day, and then maintain this level for at least another 4 days. In addition, it reached this activity peak (968 U/L) quicker at 28°C on the third day, however it could not maintain this level for as long. Even at 10°C, laccase activity was able to reach 600 U/L on the fourth day. The temperature property of laccase from Ch2-2 corresponding to the host fungus will assign it a more widespread application value.

### Synergistic action of inducible enzymes from *C. cladosporioides* Ch2-2 during the degradation of bagasse

In order to investigate the biomass degradation capability of *C. cladosporioides* Ch2-2, the main composition of its lignocellulosic enzymes system and the relationship between biodegradation and secretion regularity of enzymes, we tracked the changes of lignocellulosic enzyme activities and relevant component degradation rates during the bio-pretreatment and saccharification process of bagasse. It was found that, in addition to laccase, a variety of other inducible lignocellulosic enzymes could be produced by Ch2-2 including cellulase, xylanase and MIP, which was able to deconstruct lignin through oxidation without Mn^2+^. In the present study LiP activity was not detected. The secretion pattern of these enzymes was closely linked to the composition and structure of lignocellulose, which could cause obvious degradation rate changes of corresponding components.

**Figure 2:**
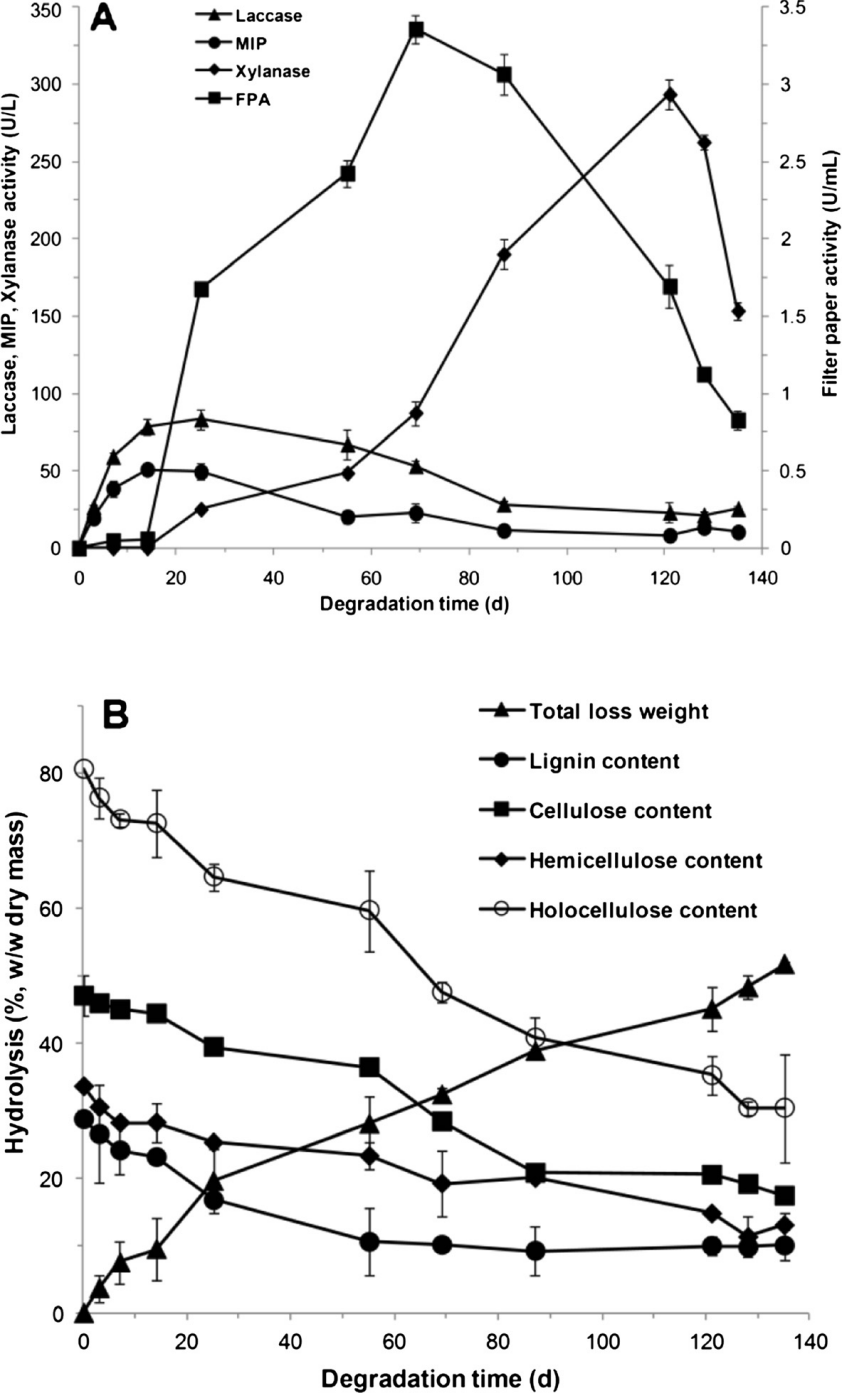
**Inducible lignocellulosic enzymes (laccase, MIP, cellulase and xylanase) (A) and changes of lignocellulose component contents (B) during bagasse degradation by** ***C. cladosporioides*** **Ch2-2 at 28°C** **.**

It was found that the activities of ligninase (laccase and MIP) were growing at a rapid rate from the beginning, following the fast degradation of lignin (Figure 2A, B). On day 14, MIP reached the maximum (51.0 U/L) and laccase also achieved its high activity level (83.2 U/L), which was sustained until day 25, further accelerating the degradation rate of lignin. During the first 25 days, the lignin degradation percentage was the highest (41.8%) as compared with that of other components, while the degradation percentages of cellulose and hemicellulose were 16% and 24.7%, respectively. After that, the activities of laccase and MIP showed a declining trend leading to a slow degradation rate of all the three components simultaneously until day 55 when the lignin content had almost reached the stable value. In contrast, cellulose began to be markedly reduced along with the slow decline of hemicellulose when the filter paper activity (FPA) was close to the peak (Figure 2A, B). When FPA was at its minimum, the cellulose content became almost stable. In accordance with the degradation regularity of cellulose, the peak of xylanase activity on day 120 (293.5 U/L) corresponded with the increased degradation rate of hemicellulose. After 135 days the total weight loss of bagasse was 51.6%, in which the losses of lignin, cellulose and hemicellulose were 65.5%, 62.8% and 61.5%, respectively.

These results indicated that multiple lignocellulosic enzymes of Ch2-2 were an efficient natural synergy system with a clear selective secretion pattern, which was induced by the specific components of bagasse. Even more importantly, it was found that the degradation rate of lignin limited that of the holocellulose to some extent.

### Ligninase purification and properties

The important role of *C. cladosporioides* Ch2-2 ligninase during the degradation process was mentioned above, which was crucial in order for cellulases and hemicellulases to carry out their roles sufficiently. In order to further analyze the ligninase characteristic, laccase and MIP were purified and studied.

**Figure 3:**
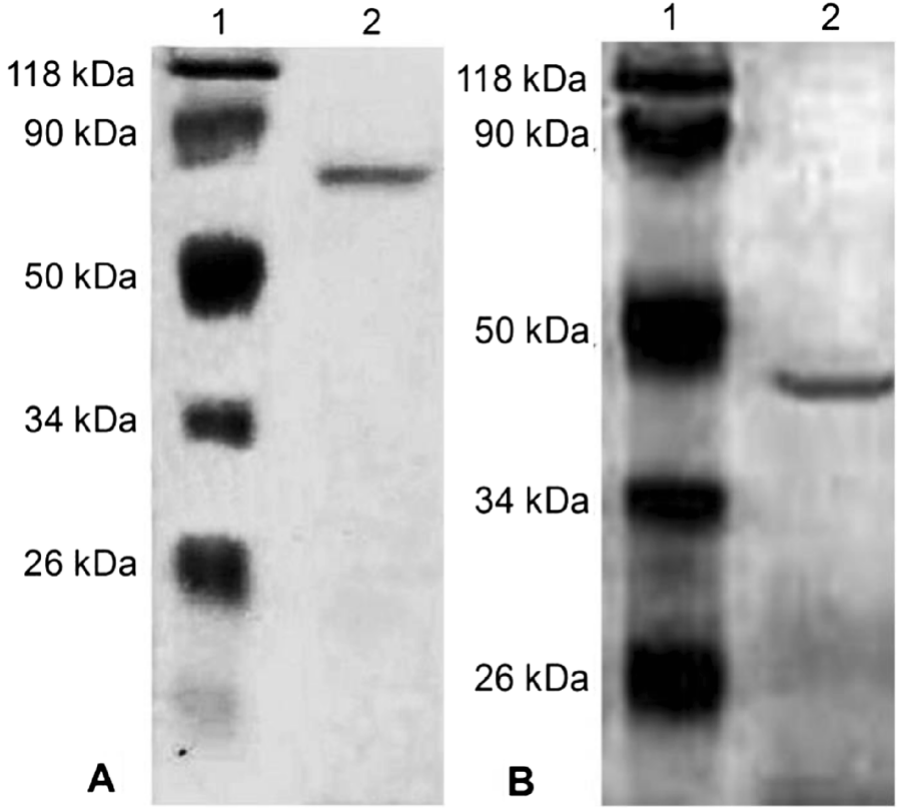
**SDS-PAGE of purified laccase and MIP from** ***C. cladosporioides*** **Ch2-2. A**. Lane 1: Molecular mass markers; Lane 2: Purified laccase. **B**. Lane 1: Molecular mass markers; Lane 2: Purified MIP.

**Figure 4:**
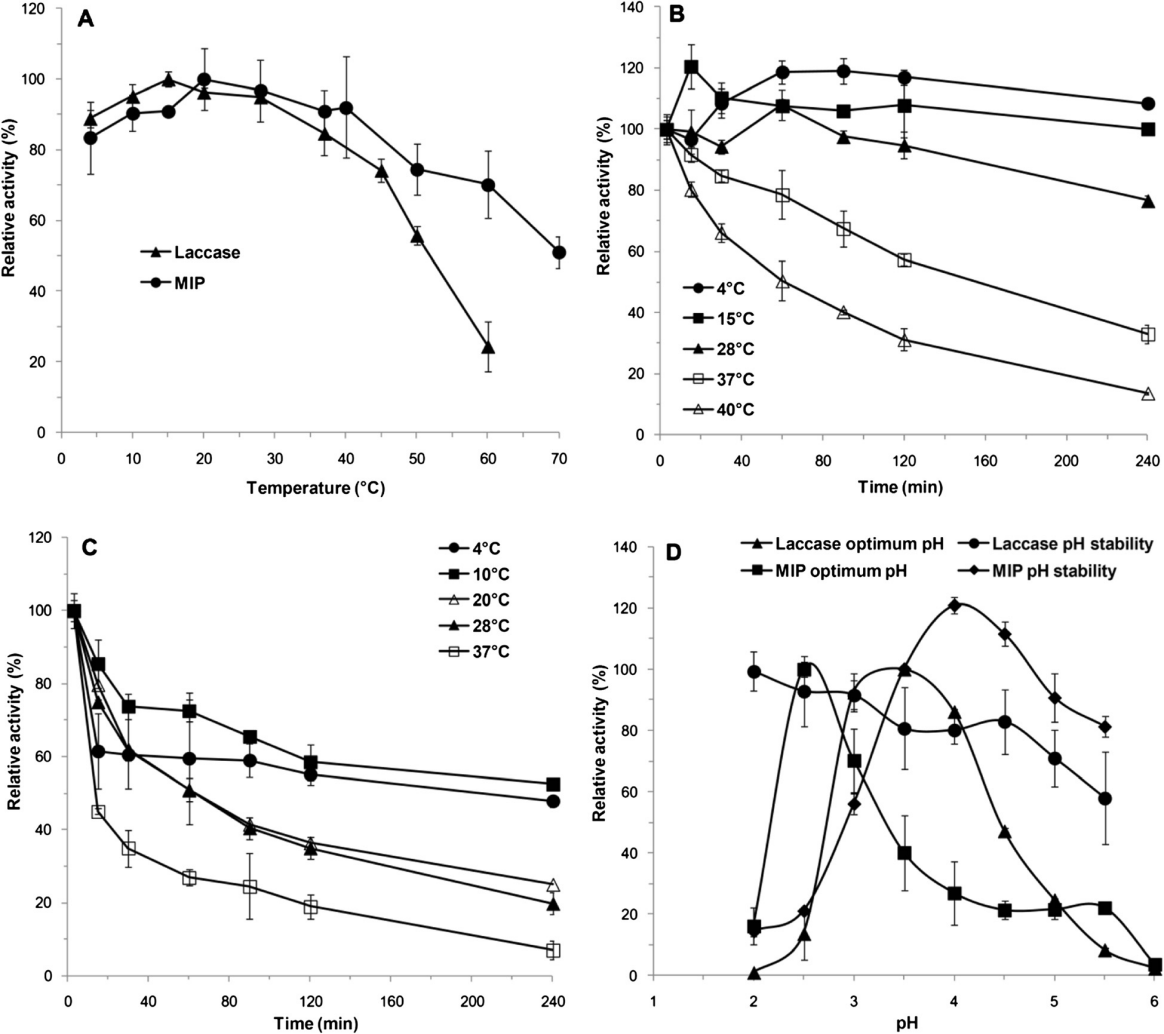
**Effects of pH and temperature on the activity and stability of purified laccase and MIP from** ***C. cladosporioides*** **Ch2-2. A**. Effects of temperatures on laccase and MIP activity. **B**. The thermostability of laccase. **C**. The thermostability of MIP. **D**. Optimal pH and pH stability of laccase and MIP. The residual activity was monitored, and the maximum activity was defined as 100% **(A, D)** or initial activity was defined as 100% **(B, C)**. Values shown were the mean of the average of three experiments.

The molecular weight of laccase was about 70 kDa as measured by SDS-PAGE (Figure 3A). Its optimal temperature and pH were 15°C and pH 3.5, respectively (Figure 4A, D). It was stable at a very wide temperature and pH range of 4 to 40°C and pH 2.0 to 5.5 (Figure 4B, D). Using ABTS as a substrate, the *K*_m_ and *V*_max_ for laccase were 2.8 μM and 57.9 μmol/min/mg under the optimal conditions. Laccase activity was inhibited by 2 mM of Fe^2+^ (5.36%), SDS (27.2%) and was slightly inhibited by EDTA (74.6%) at a concentration of 10 mM, and essentially unaffected by 2 mM of EDTA (98.0%), 10 mM of Cu^2+^ (105%) and Al^3+^ (104%).

The molecular weight of purified MIP from Ch2-2 was about 47 kDa (Figure 3B). Its optimal temperature and pH were 20°C and pH 2.5 respectively (Figure 4A, D), indicating the low temperature adaptation. It was also stable at a wide temperature and pH range of 4 to 28°C and pH 2.0 to 5.5 (Figure 4C, D). Using DMP as a substrate without H_2_O_2_, the *K*_m_ and *V*_max_ of MIP were 6.9 μM and 38.3 μmol/min/mg under optimal conditions. The MIP activity was 100% depressed by 2 mM of Fe^2+^ and essentially unaffected by EDTA (97.7%) at a concentration of 10 mM.

From enzymatic characteristics of purified enzymes, we found that both laccase and MIP from Ch2-2 exhibited substantially wide thermal adaptability in a mesophilic and psychrophilic environment, and MIP exhibited a special property that correlated with functional mechanism.

### Hydrolysis of biomass by enzymes preparation from *C. cladosporioides* Ch2-2

**Figure 5:**
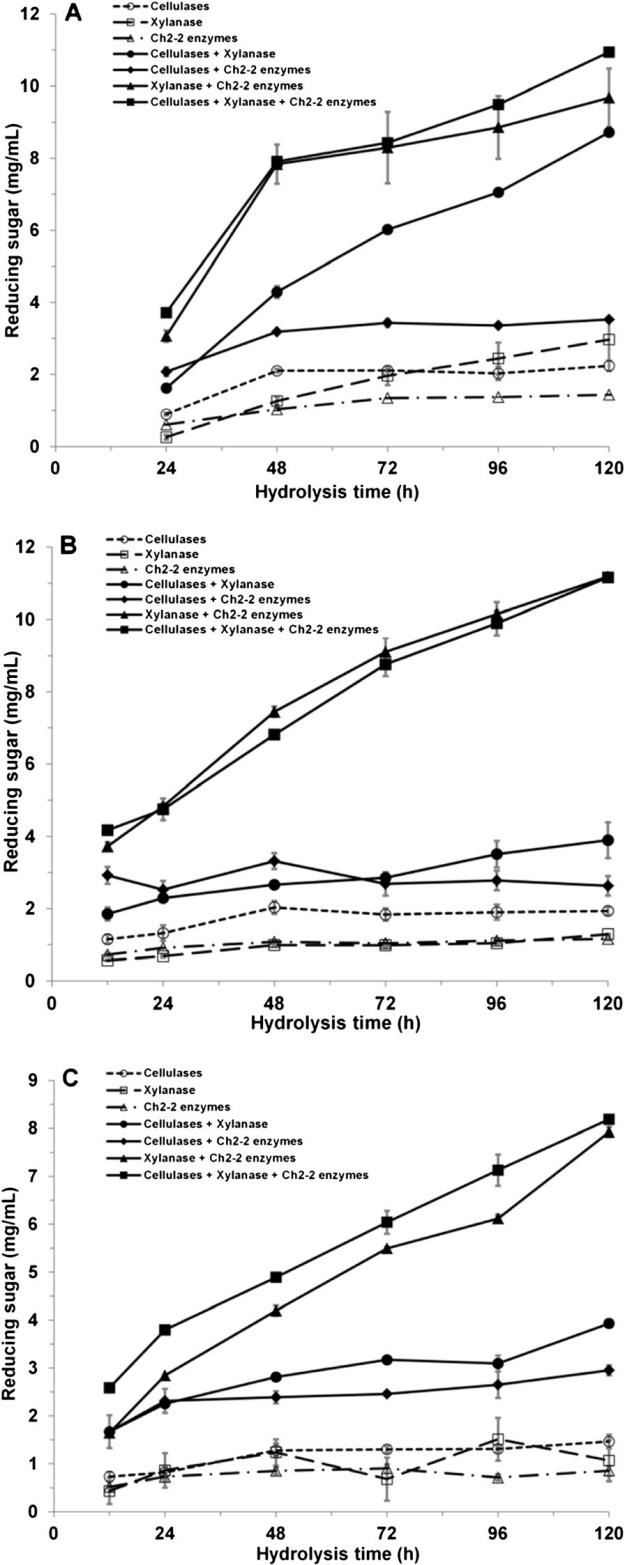
**Improved production of reducing sugars from milled Jerusalem artichoke stalks by synergy of Ch2-2 enzymes and commercial cellulase or xylanase. A**. Hydrolysis by binary (commercial cellulases/xylanase, commercial cellulases/Ch2-2 enzymes and commercial xylanase/Ch2-2 enzymes) or ternary mixtures (commercial cellulases/xylanase/Ch2-2 enzymes) at 15°C for 24 hours and then 50°C for 96 hours. **B**. Hydrolysis by binary or ternary mixtures at 28°C for 120 hours. **C**. Hydrolysis by binary or ternary mixtures at 15°C for 120 hours. Substrate control: milled Jerusalem artichoke stalks without enzymes. Enzyme control: reactions with each enzyme (commercial cellulases, commercial xylanase or Ch2-2 enzymes) alone.

According to the properties of purified laccase and MIP, synergy between Ch2-2 crude enzymes and commercial cellulase or xylanase in converting milled Jerusalem artichoke stalks into reducing sugars was assessed under different temperatures. If it was treated at 15°C for one day according to the optimum temperature of ligninase, and then at 50°C for four days in order to be fit for the commercial cellulase and xylanase, synergy was exhibited in all four types of mixture (Figure 5A). The degree of synergism (DS) between Ch2-2 enzymes and commercial xylanase reached the maximum value of 3.4 on day 2, which was followed by the substantial increase of reducing sugars (7.8 mg/mL). However, the highest reducing sugars yield was obtained by the mixture containing Ch2-2 enzymes, commercial cellulase and xylanase together on day 5 (10.9 mg/mL), which was much higher than using any enzyme alone. Therefore, it was not due to the additive effect.

We also investigated the interaction at ambient temperatures of 28°C and 15°C, respectively (Figure 5B, C). It was seen that the DS between Ch2-2 enzymes and commercial xylanase almost reached the maximum value of 4.7 at 28°C on day 4 and then reached a high reducing sugars yield of 10.1 mg/mL later on, which was even higher than that obtained at 50°C (8.9 mg/mL). During the whole hydrolysis process at 28°C, the sugars yield obtained by the mixture of Ch2-2 enzymes and commercial xylanase kept almost at the same level as that obtained by the three enzymes combined. Furthermore, the interaction also showed cold-adapted synergistic effect at 15°C with a maximum DS of 4.1 on day 5. The reducing sugars production could be improved 295% through synergistic effect between Ch2-2 enzymes and commercial xylanase on day 5 (7.9 mg/mL), which was not greatly affected by the low temperature.

**Figure 6:**
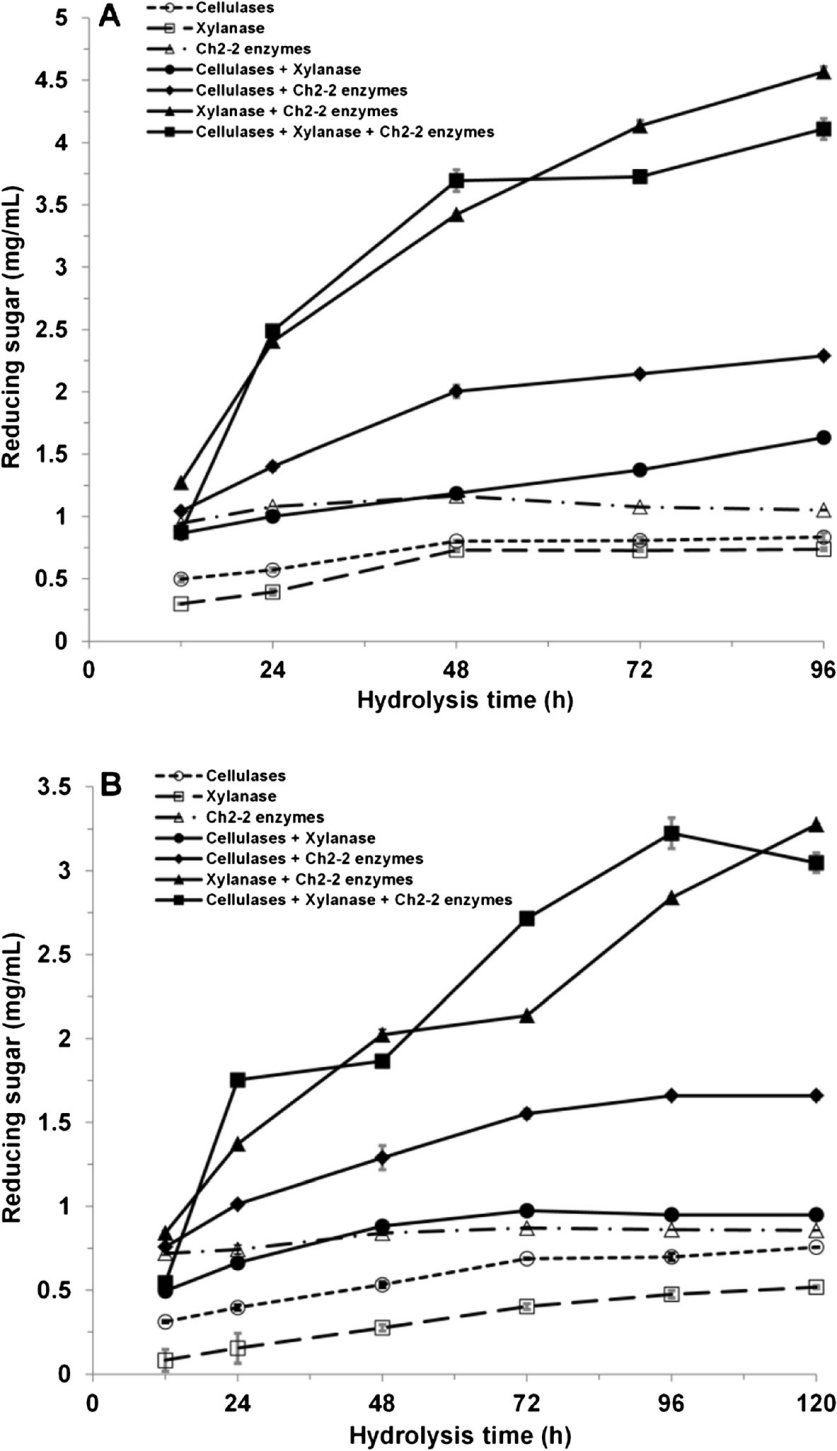
**The changes of reducing sugar production from milled Jerusalem artichoke stalks by synergy of Ch2-2 enzymes and reduced commercial cellulase or xylanase. A**. Hydrolysis by binary (commercial cellulases/xylanase, commercial cellulases/Ch2-2 enzymes and commercial xylanase/Ch2-2 enzymes) or ternary mixtures (commercial cellulases/xylanase/Ch2-2 enzymes) at 28°C for 96 hours. **B**. Hydrolysis by binary or ternary mixtures at 15°C for 120 hours. Substrate control: milled Jerusalem artichoke stalks without enzymes. Enzyme control: reactions with each enzyme (commercial cellulases, commercial xylanase or Ch2-2 enzymes) alone.

The reducing sugar production from milled Jerusalem artichoke stalks and the synergy effect between enzymes were further assessed under 28°C and 15°C when the loading of commercial cellulase and xylanase were reduced proportionally to one fifth; 0.05 g cellulase/g substrate (25 mg protein/g cellulose) and 0.3 g xylanase/g substrate (26 mg protein/g cellulose), respectively (Figure 6A, B). The results showed that the best synergistic effect still occurred between Ch2-2 enzymes and commercial xylanase, with a DS of 2.6 and a reducing sugar level of 4.6 mg/mL on day 4 (28°C), while the concentration of reducing sugar reached 4.1 mg/mL through the ternary mixtures. Cold-adapted synergistic effect at 15°C between Ch2-2 enzymes and commercial xylanase remained maximum with a DS of 2.4 and a reducing sugar level of 3.3 mg/mL.

The results indicate that crude enzymes mixture containing ligninase from *C. cladosporioides* Ch2-2 was able to enhance hydrolysis of other polysaccharide hydrolases significantly over a wide temperature range, which has a promising prospect for application in biomass degradation and high-value energy production at ambient temperature. It could provide a novel guidance for the design and operation of lignocellulosic enzyme cocktails with efficient synergistic effect.

## Discussion

In the overall process of lignocellulosic ethanol production, the cost of enzymes accounts for around 50 to 60% [26], with energy consumption being close behind [27]. Energy is mainly consumed in biomass pretreatment, saccharification and fermentation and so on. The expensive cost of enzymes and energy limits the market of cellulosic ethanol, and so a dramatic reduction in enzyme and energy use is extremely necessary to enhance competitiveness. In this study, psychrophilic *C. cladosporioides* Ch2-2 was found to be effective in bio-pretreatment and saccharification on bagasse. At the same time, synergy of crude enzymes from Ch2-2 with commercial xylanase greatly enhanced saccharification of milled Jerusalem artichoke stalks at 28°C. In view of the application temperature, when using the most common industrial strain *Saccharomyces cerevisiae* for ethanol fermentation, saccharification and fermentation could be unified at its optimum growth temperature.

During the degradation of bagasse, both the secretion of lignocellulosic enzymes and the decrease of corresponding lignocellulose components were much more selective by fungus *C. cladosporioides* Ch2-2 than by the three white-rot fungi reported by Dong *et al*. [28]. For the same lignocellulosic substrate, different fungi possessed unique degradation strategies by producing distinct enzymes, which implied various synergistic manners and mechanisms in nature. The clear separate degradation pattern during different periods made Ch2-2 more suitable for selective delignification than white-rot fungi. At the same time, the pivotal role of ligninase was highlighted in this process. Laccase and MIP were released simultaneously first following lignin degradation at a higher rate compared to holocellulose. The degradation degree of lignin drastically affected the hydrolysis of holocellulose. On one hand, the residual lignin depressed the activity of polysaccharide hydrolases and the degradation rate of holocellulose [9,10]. On the other hand, lignin degradation could decrease non-productive adsorption of polysaccharide hydrolases and accelerate hydrolysis [29].

Ligninase from Ch2-2 showed the adaptability to moderate and low temperatures, corresponding to the growth characteristics of the host fungus. The purified laccase and MIP were stable in a wide temperature range of 4 to 40°C and 4 to 28°C respectively, which verified that Ch2-2 was a cold-adapted lignin-degrading fungus. It greatly expanded the application scope of Ch2-2 and its enzymes, including straw returning during intermittent period of farming besides cellulosic ethanol production at room temperature. In a previous report, psychrophilic enzymes produced by cold-adapted microorganisms most often displayed high thermosensitivity [30]. Higher temperatures (>20°C) typically led to low densities of cells and poor extracellular enzyme production, although shortening the generation time. However, for Ch2-2, the dry cell weight and laccase activity always exhibited high levels whether at 15°C or at 28°C. The thermal stable range for laccase and MIP from Ch2-2 performed the best out of the psychrophilic enzymes reported. Recently, many researchers have obtained thermostable polysaccharide hydrolases with good hydrolysis ability from thermophilic strains [31]. The cellulase mixture from thermophilic fungi was often evaluated at temperature 35 to 65°C [32], however, it was uneconomical in operation because of higher demands on devices and power consumption. Psychrotolerant enzymes have considerable application prospects in biological refining industry. At present, there are only three psychrophilic *C. cladosporioides* reported, which were obtained from wood samples in Antarctica producing more endoglucanase activity at 4°C than at 15°C after 10 days cultivation. There were no further reports about their degradation and wider mesophilic adaptabilities.

Like the digestion of novel mechanism of thermophilic *Caldicellulosiruptor bescii* CelA [33], psychrophilic enzymes from Ch2-2 provided the possibility of exploring a new synergy mechanism. At present, it is not clear what role MIP plays in lignin degradation. The molecular weight of laccase was higher than that reported for MnP, which made it hard to access occluded lignin in biomass. Various low molecular weight electron transfer agents such as 1-hydroxybenzotriazole (HOBT) were usually needed by laccase to catalyze ligninolysis [34]. While MnP produced small diffusible strong oxidants that could penetrate the substrate, it needed Mn^2+^ induction and H_2_O_2_ to work [35,36]. However, psychrophilic MIP from Ch2-2 plays its role without Mn^2+^ and H_2_O_2_ while being essentially unaffected by EDTA and completely depressed by Fe^2+^, which has not been previously reported. Therefore, future studies should be directed at elucidating the mechanism that MIP serves in the ligninolytic and synergistic hydrolysis system.

Onozuka R-10 was one kind of expensive commercial cellulase mixture used in cellulose hydrolysis as reported [35], which was made of a variety of hydrolytic enzymes including endo-1, 3-β-D-glucanase, β-glucosidase, xylanase, pectinase, protease and α-amylase. The cost of commercial xylanase from *Aspergillus clavatus* used in this study accounted for about one-fifteenth that of the Onozuka R-10. Their optimum range of temperature and pH were 40 - 50°C and pH 4.0 - 5.0. During the hydrolysis of Jerusalem artichoke stalks with different enzymes combination, interaction between Ch2-2 crude enzymes and commercial xylanase exhibited maximum synergistic effect at 15, 28 and 50°C, which could take the place of an expensive commercial cellulase mixture in application. The reducing sugars yield received through the mixture of Ch2-2 enzymes and commercial xylanase here at 28°C (482.9 mg/g substrate) was much higher than that using a fungal-bacterial cocktail of pure strains at 30°C (165.2 mg/g substrate) within 24 hours [37]. Acquiring a considerable sugars yield from biomass at normal temperature is also profitable in providing a carbon source for microalgae lipid production by heterotrophic growth of microalgae, which often achieves maximum performance at meso-low temperatures [38]. These results provide novel information for constructing an efficient and easy-operating composite lignocellulosic enzymes mixture. In this way, the saccharification for fermentable sugars can be maximized and the bioethanol yield can be increased even at normal and low temperatures to meet the demand of bio-refinery.

## Conclusions

Cold-adapted fungus *C. cladosporioides* Ch2-2 possesses a natural synergistic lignocellulosic enzymes system suitable for the selective delignification and efficient saccharification of biomass. Purified laccase and MIP from Ch2-2 had a wide thermal adaptability in mesophilic and low temperatures corresponding to the host fungus. MIP provided a new enzyme property in cooperation with other enzymes during biodegradation. Hydrolysis of lignocellulosic substrate was severely enhanced by synergy between crude Ch2-2 enzymes and cheap commercial xylanase, which could provide a substitute for current expensive commercial cellulase mixtures and reach a higher reducing sugars yield. The new cocktail of Ch2-2 enzymes and commercial xylanase enabled consistency of hydrolysis and fermentation at ambient temperatures. This study will provide a novel perspective of lignocellulosic enzyme construction and psychrophilic enzymes application in biotechnology, including waste treatment, high-value renewable biofuel production and bioremediation at ambient temperatures.

## Materials and Methods

### Fungus thermal adaptability

Seed liquid of *C. cladosporioides* Ch2-2 was inoculated to GPY medium (2% glucose, 0.5% peptone, 0.2% yeast extract, 0.2% KH_2_PO_4_, 0.05% MgSO_4_/7H_2_O, pH 5.5) cultured for 7 days at 10 to 28°C. The dry weight of mycelia was determined separately.

At the same time, the fungus solution activated in different temperatures was transferred into the optimized medium (20% potato juice, 2% sucrose, 5% wheat-bran) for laccase production and activity detection.

### Enzymatic degradation

5 g of dry bagasse was added in 150 mL conical flasks and moistened with 30 mL liquid Czapek culture medium [28]. After autoclaving for 1 hour at 121°C, each flask was inoculated with 5 mL seed liquid of *C. cladosporioides* Ch2-2 and incubated at 28°C [39]. In all cases, bagasse samples were treated identically but the sample without fungal inoculation served as a control. The samples were regularly tested to determine dry weight, chemical composition and enzyme activities using methods previously described [40]. All experiments were performed in triplicate.

### Enzymes purification

Two major ligninase components, laccase and MIP, were isolated and purified from the fermentation broth of *C. cladosporioides* Ch2-2 using the techniques of (NH_4_)_2_SO_4_ precipitation, anion and cation exchange chromatography. The purity of the enzymes was confirmed by SDS-PAGE [41].

The supernatant of three-day-old culture was collected by suction filtration and centrifugation at 7,155 g for 10 minutes at 4°C. Solid ammonium sulfate was added to this supernatant up to 85% saturation and the resulting precipitate was collected by centrifugation at 11,180 g for 15 minutes. The precipitate was re-dissolved in 2 mL of 10 mM sodium acetate buffer, pH 4.5, and dialyzed overnight. Precipitated material was removed by centrifugation at 800 g for 10 minutes and the supernatant was applied to a column (2.5 × 20 cm) of DEAE-Sephadex A 50. The enzyme was eluted gradually with 10 mM pH 8.0 Tris buffer with NaCl of 300 mM, 500 mM and 1 M at a flow rate of 1.2 mL/min. The fraction containing maximum laccase or MIP activity was collected and desalted by dialysis.

The collection with laccase activity was applied to a column (1 × 40 cm) of Sephadex G-75. The enzyme was eluted with 10 mM pH 7.0 phosphate buffer with NaCl of 0.15 M at a flow rate of 0.32 mL/min. The fraction containing maximum laccase activity was collected for further analysis [18].

The first purification step of collection with MIP activity was the same as laccase. Then it was applied to a column (1 × 17 cm) of CM-Sephadex C 50. The enzyme was eluted gradually with 10 mM pH 5.7 acetate buffer with 50 mM NaCl at a flow rate of 0.2 mL/min. The third step was as the first one with the Sephadex G-75 column (1.5 × 60 cm). The fraction containing maximum MIP activity was collected for further analysis.

### Enzymes properties

The optimum pH for laccase and MIP were determined by incubation at various pH conditions (pH 2.0 to 6.0) at 28°C for 10 minutes in 50 mM sodium acetate buffer and malonic acid-sodium malonate buffer, respectively. The optimum temperature for the enzyme activity was determined by standard assay ranging from 4 to 70°C in the same buffer as above at pH 4.5. The results were expressed as relative activity to the value obtained at either optimum temperature assay or optimum pH assay.

The pH stability of the enzymes were determined by measuring the remaining activity after incubating the enzymes at 28°C for 2 hours in 50 mM sodium acetate buffer and malonic acid-sodium malonate buffer respectively from pH 2.0 to 5.5. To determine the effect of temperature on the stability of the enzymes in the same buffer as above (pH 4.5), the remaining activity after incubating the enzymes for 4 hours at 4 to 40°C was measured. The activity of the enzyme obtained at either optimum temperature or optimum pH was defined as 100%.

Kinetic constants of laccase and MIP were determined by measuring the initial rates at various ABTS and DMP concentrations respectively under standard reaction conditions. The *K*_*m*_ and *V*_*max*_ were determined at 28°C by recording the effect of various concentrations (10 to 40 mmol/L) of ABTS and DMP separately [42].

The effects of metal ions and chemical reagents on enzyme activity of purified laccase and MIP were determined. The enzyme was incubated with each reagent for 2 hours at 28°C before the addition of the substrate to initiate the enzyme reaction. The activity of the enzyme without adding chemical reagents or metal ions was defined as 100%.

### Enzymatic hydrolysis

The freeze-drying powder of crude enzymes from *C. cladosporioides* Ch2-2 was used in different combinations at a loading of 0.1 g/g substrate. The commercial cellulase (Onozuka R-10, Merck) and xylanase (BioRoYee) were assessed at a loading of 0.25 g/g substrate and 1.5 g/g substrate, respectively. The loading of 0.05 g cellulase/g substrate (25 mg protein/g cellulose) and 0.3 g xylanase/g substrate (26 mg protein/g cellulose) were also assessed. The hydrolysis assays were carried out at 1% (w/v) solid loading of grinding Jerusalem artichoke stalk in sodium acetate buffer (50 mM, pH 4.5) in a 2 mL total volume. The reaction mixtures were mechanically shaken in an orbital shaker incubator at 15°C for 24 hours and then at 50°C for another 96 hours. The same reactions were individually performed at 15°C and 28°C up to 120 hours. The hydrolysis was terminated by boiling the reaction mixture at 100°C for 10 minutes to inactivate the enzymes. The supernatants collected after centrifugation at 9,300 g for 10 minutes for the measurement of reducing sugars. Blanks were run at the same time by incubating the substrates without enzymes. Reactions with each enzyme only were used as bases. All hydrolysis experiments were performed in triplicate and mean values and standard deviations are presented. The degree of synergism was calculated by the method described earlier [4].

### Analytical methods

The fungal biomass was detected by using the oven-drying method [25]. The samples cultured at different temperatures were first vacuum-suction filtrated. Collected mycelia were washed by distilled water and dried for 24 hours at 50°C in an oven until the weight was constant.

FPA was measured spectrophotometrically based upon the color reaction between the degradation products (glucose) and DNS (3,5-dinitrosalicylic acid) [43]. The enzyme sample was mixed with 50 mM citrate-phosphate buffer (pH 4.5) and Whatman filter paper. After incubation at 30°C for 60 minutes, the mixture was boiled with DNS for 5 minutes and the absorbance was measured at 520 nm. One activity unit was defined as the amount of cellulase needed to release 1 μmol of glucose equivalent sugar per minute.

Xylanase activity was also assayed based upon the color reaction between the degradation products (xylose) and DNS. The enzyme sample was mixed with 50 mM citrate-phosphate buffer (pH 4.5) and 1% Birchwood xylan. After incubation at 30°C for 15 minutes, the mixture was boiled with DNS for 5 minutes and the absorbance was measured at 520 nm [43]. One activity unit was defined as the amount of xylanase needed to release 1 μmol of xylose equivalent sugar per minute.

Laccase activity was determined using ABTS (0.5 mM) as the reducing substrate [44]. The enzyme sample was mixed with 50 mM sodium acetate buffer (pH 4.5) and ABTS. After incubation at 30°C for 10 minutes, the mixture was transferred into an ice-water bath to stop the reaction, and the absorbance was measured at 420 nm. One activity unit was defined as the amount of laccase required to oxidize 1 μmol of substrate per minute.

MIP activity was detected by monitoring the oxidation of DMP in the absence of Mn^2+^ and H_2_O_2_ [12]. The reaction mixture contained enzyme sample and 2 mM DMP in 50 mM malonic acid-sodium malonate buffer (pH 4.5). After incubation at 30°C for 10 minutes, the absorbance was measured at 468 nm. One activity unit was defined as the amount of enzyme that produced 1 μmol product per minute.

LiP activity was assessed by determining the oxidation of Azure Blue using H_2_O_2_ [45]. The assay system contained enzyme sample, 32 μM Azure Blue and 100 μM H_2_O_2_ in 50 mM sodium tartrate buffer (pH 4.5). After incubation at 30°C for 10 minutes, the absorbance was measured at 651 nm. One activity unit was defined as the amount of enzyme that produced 1 μmol product per minute.

The quantitative analysis of chemical compositions of bagasse including lignin, cellulose and hemicellulose were performed by methods described earlier [46,47]. The reducing sugars were quantified by the DNS method [48]. The estimation of protein content was determined according to the method described in Lowry *et al*. [49]. All experiments were performed in triplicate and mean values and standard deviations are presented.

## Abbreviations

ABTS: 2, 2′-Azino-bis-(3-ethylbenzothiazoline-6-sulfonic acid) diammonium salt
DMP: Dimethyl phthalate
DNS: 3,5-dinitrosalicylic acid
DS: Degree of synergism
FPA: Filter paper activity
HOBT: 1-hydroxybenzotriazole
LiP: Lignin peroxidase
MIP: Manganese-independent peroxidase
MnP: Manganese peroxidases
SDS: Sodium dodecyl sulfate
SDS-PAGE: SDS-polyacrylamide gel electrophoresis
